# Pan-American Medical Congress

**Published:** 1893-06

**Authors:** 


### PAN-AMERICAN MEDICAL CONGRESS.

CINCINNATI, OHIO, May 24, 1893.

*Editor Daniel's Texas Medical Journal:*

The date of the Pan-American Medical Congress is near at hand.

The prospects for a brilliantly successful celebration of this important medical event are most encouraging.

The Congress will be opened by the President of the United States on September the fifth, and an official reception at the White House will be given on a date not yet determined.

The invitation extended by the National Government to the Pan-American countries has been uniformly accepted, and many highly distinguished delegates will attend officially. The presence of a number of distinguished European guests is assured. The scientific papers secured for the various sections are numerous and important, so that the proceedings of the Congress will certainly be highly interesting and valuable.

It may be safely asserted that the volumes of Transactions will alone far exceed in value the amount of registration fee. The Committee of Arrangements will provide ample accommodation for all portions of the work of the Congress; and the entertainments proposed are unusually attractive. It is absolutely indispensable that an estimate should be now formed of the number likely to attend. It is confidently hoped that a very large representation of our profession will be present on an occasion of such practical and historic interest.

Will you kindly proceed at once to ascertain by personal enquiry and solicitation how many of your professional constituents propose to attend.

You are hereby officially requested to secure as many registrations as possible, and to have the registration fees forwarded to the Treasurer, Dr. A. M. Owen, 507 Upper First street, Evansville, Indiana, who will promptly return membership tickets therefor. Yours sincerely,

CHARLES A. L. REED,
By the Executive Committee, Secretary-General.
Approved:
WILLIAM PEPPER, President.

---

The Twenty-third Quarterly Meeting of the Austin District Medical Society will be hold in Austin, Texas, Thursday, June 22, 1893. You are invited to be present, and participate in the following programme:

1. "A Report of a Case of Bright's Disease of 30 Years' Standing," by Dr. W. E. Shelton; discussion opened by Dr. R. Atkinson and Dr. J. Cummings.

2. "The Treatment of Salpingitis without Operation," by Dr. W. J. Mathews; discussion opened by Dr. B. E. Hadra and Dr. J. W. McLaughlin.

3. "Some Observations on Puerperal Eclampsia," by Dr. S. Cunningham; discussion opened by Dr. C. O. Weller and Dr. S. M. Morris.

4. "The Keely Cure—Its Ethical Status, etc.," by Dr. F. E. Daniel; discussion opened by Dr. R. M. Swearingen and Dr. W. T. Richmond.

5. "Spinal Concussion and John Eric Erichson's Book," by Dr. D. R. Wallace; discussion opened by Dr. T. D. Wooten and Dr. G. W. Christian.

R. P. TALLEY, President.
T. J. BENNETT, Secretary.

---

**University Regents.**—Judge E. A. Cowart, of Dallas, has been appointed on the Board of Regents, to fill the vacancy occasioned by the resignation of Judge Simkins.

